# Machine Learning-Based Measurement of Regional and Global Spinal Parameters Using the Concept of Incidence Angle of Inflection Points

**DOI:** 10.3390/bioengineering10101236

**Published:** 2023-10-23

**Authors:** Thong Phi Nguyen, Ji-Hwan Kim, Seong-Ha Kim, Jonghun Yoon, Sung-Hoon Choi

**Affiliations:** 1 Department of Mechanical Engineering, BK21 FOUR ERICA-ACE Center, Hanyang University, 55 Hanyangdaehak-ro, Sangnok-gu, Ansan-si 15588, Gyeonggi-do, Republic of Korea; npthong2511@hanyang.ac.kr; 2 Department of Mechanical Engineering, Hanyang University, 55 Hanyangdaehak-ro, Sangnok-gu, Ansan-si 15588, Gyeonggi-do, Republic of Korea; 3 Department of Orthopedic Surgery, Hanyang University College of Medicine, 222 Wangsimni-ro, Seongdong-gu, Seoul 04763, Republic of Korea; kimscien@hanyang.ac.kr (J.-H.K.); seong02ha13@hanyang.ac.kr (S.-H.K.); 4 AIDICOME Inc., 221, 5th Engineering Building, 55 Hanyangdaehak-ro, Sangnok-gu, Ansan-si 15588, Gyeonggi-do, Republic of Korea

**Keywords:** machine learning, convolutional neural network, sagittal alignment, pelvic incidence, incidence angle of inflection points, lumbar lordosis, cervical lordosis

## Abstract

This study delves into the application of convolutional neural networks (CNNs) in evaluating spinal sagittal alignment, introducing the innovative concept of incidence angles of inflection points (IAIPs) as intuitive parameters to capture the interplay between pelvic and spinal alignment. Pioneering the fusion of IAIPs with machine learning for sagittal alignment analysis, this research scrutinized whole-spine lateral radiographs from hundreds of patients who visited a single institution, utilizing high-quality images for parameter assessments. Noteworthy findings revealed robust success rates for certain parameters, including pelvic and C2 incidence angles, but comparatively lower rates for sacral slope and L1 incidence. The proposed CNN-based machine learning method demonstrated remarkable efficiency, achieving an impressive 80 percent detection rate for various spinal angles, such as lumbar lordosis and thoracic kyphosis, with a precise error threshold of 3.5°. Further bolstering the study’s credibility, measurements derived from the novel formula closely aligned with those directly extracted from the CNN model. In conclusion, this research underscores the utility of the CNN-based deep learning algorithm in delivering precise measurements of spinal sagittal parameters, and highlights the potential for integrating machine learning with the IAIP concept for comprehensive data accumulation in the domain of sagittal spinal alignment analysis, thus advancing our understanding of spinal health.

## 1. Introduction

The human spine exhibits a characteristic sagittal plane alignment known as lordosis in both the lumbar and cervical regions. This alignment is essential for maintaining an upright posture and facilitating a gait that enables the unrestricted use of both arms [1,2]. It has been well-established that maintaining proper sagittal alignment of the spine is a critical factor in the management of pain and disability in patients with adult spinal deformities [3,4]. Various sagittal angular parameters such as pelvic incidence minus lumbar lordosis, and T1 slope minus cervical lordosis have been reported as the primary parameters representing cervical and thoracolumbar deformities, respectively [5,6]. However, most spinal sagittal parameters require manual measurement by spine surgeons, which inevitably requires considerable time and effort. Moreover, this practice limits the scale of the collected data, and is insufficient for comprehensively analyzing the characteristics of spinal alignment.

While originally introduced in the 1980s, artificial intelligence is currently exhibiting rapid growth with the development of computational performance [7]. Deep learning, a subconcept of machine learning, can process a large amount of information by generating optimized weights while learning similarly to a human, and has an advantage in repetitive tasks in a short time [8]. Convolutional neural networks (CNNs) are widely used to extract features from image data, and a few studies have recently reported their application in spinal sagittal analysis. For instance, Aubert et al. [9] introduced a pioneering technique for the automated reconstruction of three-dimensional spinal structures. Their approach involved the use of a convolutional neural network (CNN) to precisely fit a statistically plausible model of the spine to medical imaging data. In a similar context, Weng et al. [10] introduced a deep learning methodology employing regression techniques to accurately estimate the sagittal vertical axis—a pivotal parameter characterizing sagittal alignment. Extending the scope of vital spinal measurements, which encompass cervical lordosis, thoracic kyphosis, pelvic incidence minus lumbar lordosis, sagittal vertical axis, and pelvic tilt, Cho et al. [11] conducted a comprehensive study utilizing the U-net architecture for precise anatomical landmark segmentation in radiographic imagery. Additionally, Wu et al. [12] proposed an innovative multiview correlation network architecture aimed at measuring the Cobb angle, primarily relying on the detection of anatomical landmarks. However, their approach did not specifically address parameters related to sagittal alignment. In the pursuit of automating the measurement of spinal alignment parameters through machine learning, Chae et al. [13] and Nguyen et al. [14,15] developed a sophisticated algorithm grounded in distributed convolutional neural networks. Notably, their algorithm demonstrated a significant correlation with manual measurements conducted by experienced spine surgeons [16].

To facilitate a more intuitive interpretation of spinal alignment, incidence angles of the inflection points (IAIPs) have been introduced as valuable parameters that depict the geometric relationships between the pelvis and the spine [17]. These inflection points, specifically defined as L1, T1, and C2, give rise to corresponding incidence angles: L1 incidence (L1I), T1 incidence (T1I), and C2 incidence (C2I). These angles represent the relationship between the extension of the pelvic tilt vector line and the perpendicular line drawn from the upper endplate of each respective vertebra. These IAIPs represent the cumulative geometric summation extending from the pelvis to each individual vertebra. They are computed as the aggregate of the slope angle at each vertebral level combined with the pelvic tilt, akin to the calculation of the pelvic incidence [18,19,20,21,22,23,24]. Therefore, in this study, the IAIPs were measured using CNN-based machine learning, and the accuracy of the thoracolumbar angular parameters derived through geometric relationships was evaluated. The hypothesis of this study was that machine learning-based measurement of global sagittal spinal parameters and estimates derived using spinal geometric equations would show high accuracy.

## 2. Materials and Methods

### 2.1. Patient Enrollment

We analyzed the whole-spine lateral radiographs of 595 patients who visited a single institution between March 2019 and August 2021. The inclusion criteria were patients aged over 20 years with mild pain and a visual analog score of 4 or less, and none of the patients had restrictions on walking for >30 min. The exclusion criteria were the patient who underwent previous instrumentation surgery of the spine, hip and knee replacement surgery, history of vertebral compression fractures, and pregnancy. All patients were radiographically imaged using a standardized protocol, 36-inch full-length film. Following the exclusion of subpar-quality images of the spinal endplates and both femoral heads, only images of superior quality, suitable for meticulous contrast and brightness examination, were retained for analysis. This study received approval from our institutional review board (2020-05-032-008).

### 2.2. Image Preparation and Measurement

**Figure 1:**
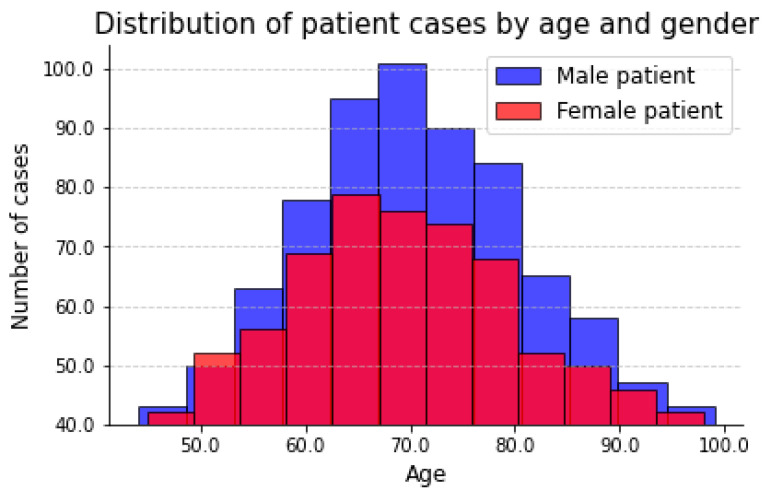
Distribution of patient cases by age and gender.

For the whole-spine standing radiographs for parameter measurement, 563 whole-spine lateral radiography sheets among the 595 high-quality images were analyzed. The distribution of patient images along age and gender is shown in the Figure 1.

Whole-spine standing radiographs were obtained with the hands naturally placed on both clavicles, without holding the supporting bar, in a standing state, facing forward, with the hip and knee joints extended as much as possible. Radiographs were obtained using an X-ray scanner with an average size of 3240 × 1080 pixels. The ratio of the training and validation sets was set at 9:1; training was performed with images from 500 patients, and the measured values were validated with images from 63 patients.

## 3. Machine Learning-Based Analysis Program and Training Process

The inception of the CNN traces back to the pioneering work of Fukushima et al. [25] in 1980, with the introduction of the ‘neocognitron’. This architectural design represents a specialized neural network engineered to discern intricate features within images, and establish the relationships between these features and desired outcomes. Comprising input, output, and multiple intermediary layers, X-ray images are initially conveyed to the network’s input layer. These intermediate layers serve the critical function of feature extraction from the input images, thereby providing valuable insights for the assessment of landmark positions, a key component of the anticipated outcomes. This function is constructed using a training process, during which the weight factors, which represent the relationship between the features and outcome, were optimized by comparing the predicted and ground truth values. The weight factor adjustment is achieved by the difference between the output $A^{j}$ from the deep learning model containing the calculated position value j for the input image and the label $Y^{j}$, created based on the actual position of the required points in the input image. This disparity is calculated using the mean absolute error as a loss function, as expressed in Equation (1), with *n* labels in the dataset. The L value then increases as the difference between $A^{j}$ and $Y^{j}$ increases.

(1) $$L=\frac{\sum_{i=1}^{n}\left|A^{j}-Y^{j}\right|}{n}.$$

**Figure 2:**
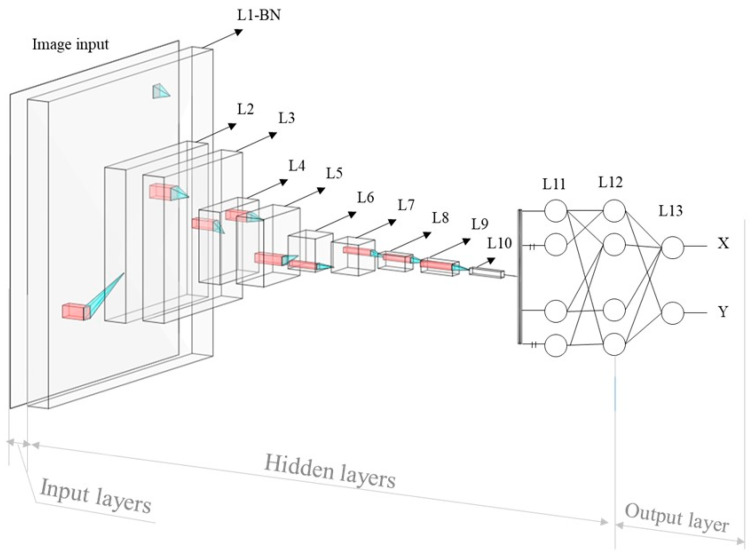
Structure of a convolutional neural network model.

The optimization of the weight factor, $w_{q}^{p}$, between the p and q nodes located in the neighboring layer is performed based on the difference from the loss function in the direction from the output to the input layer. After each training step, the weight factor $w_{p}^{q}$, is updated by $\Delta w_{p}^{q}$, which can be derived from Equation (2) with a learning rate, α, determining the learning speed of the model [26,27], where $z_{q}$ denotes the summation of the weighted input data multiplied by the weight factor, and $o_{q}$ is the output signal of the node, as shown in Figure 2.

(2) $$\Delta w_{p}^{q}=-\alpha \frac{\partial L}{\partial o_{q}}\frac{\partial o_{q}}{\partial z_{q}}\frac{\partial z_{q}}{\partial w_{p}^{q}}$$

**Figure 3:**
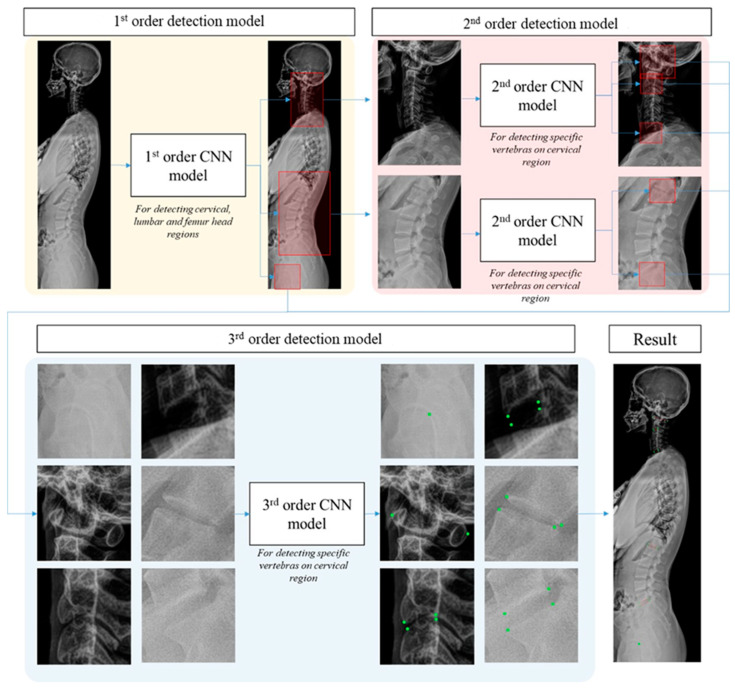
Detection procedure for required landmarks (green dot) using a decentralized convolutional neural network with 1st (yellow), 2nd (red) and 3rd (blue) CNN models.

The accurate detection of landmarks is of paramount importance in this context, as it directly influences the precision of angle parameter measurements. To address this critical need, we employed a decentralized CNN model [13,14,15,16], previously established as a robust landmark detection technique for medical images. This method offers the distinct advantage of narrowing the region of interest (ROI) on a per-order basis, effectively reducing the influence of extraneous features on results, and enhancing the diversity of the training dataset. Our proposed decentralized CNN model, as depicted in Figure 3, comprises three orders. The 1st order serves to provide a rough detection of the regions encompassing the cervical, lumbar, and hip bones. The 2nd order then refines this detection by locating specific vertebrae, while the 3rd order is tasked with the precise localization of landmarks on each identified vertebra. This multitiered approach was devised to tackle the inherent complexity of landmark detection. The required angular parameters can be measured from the detected landmarks. Each angle α consists of two vectors, $v_{\alpha_{1}}$ and $v_{\alpha_{2}}$, which are shaped by two pairs of landmarks $(P_{1}^{\alpha_{1}}$, $P_{2}^{\alpha_{1}})$, $(P_{1}^{\alpha_{2}}$, $P_{2}^{\alpha_{2}})$. Therefore, the angle was calculated using Equation (3), as follows:

(3) $$\alpha =\arccos\left(\frac{\vec{P_{1}^{\alpha_{1}}P_{2}^{\alpha_{1}}}.\vec{P_{1}^{\alpha_{2}}P_{2}^{\alpha_{2}}}}{\left|\vec{P_{1}^{\alpha_{1}}P_{2}^{\alpha_{1}}}\right|\left|\vec{P_{1}^{\alpha_{2}}P_{2}^{\alpha_{2}}}\right|}\right)$$

## 4. Measurement of Parameters after the Training Process

**Figure 4:**
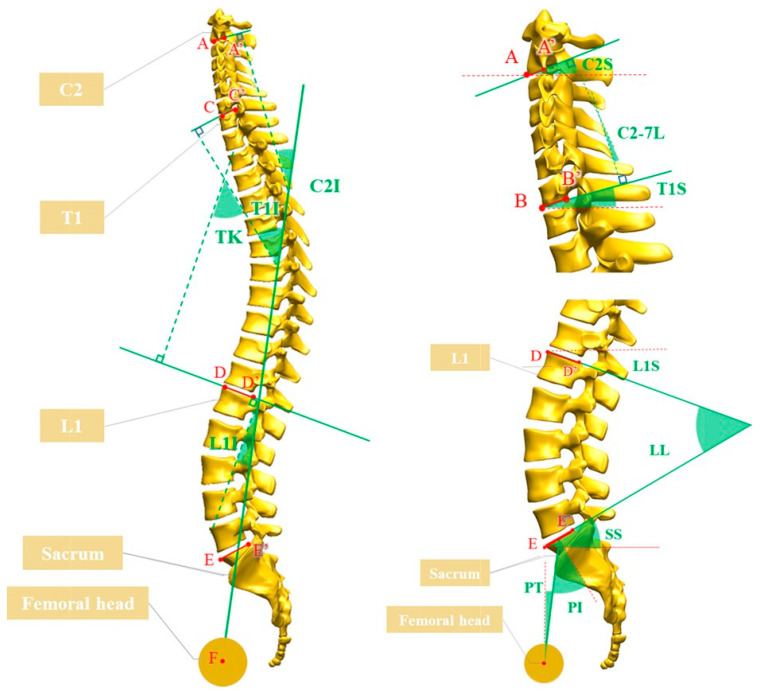
Schematic drawings of radiographic parameters of spinal sagittal alignment. Lumbar lordosis = L1 incidence − pelvic incidence; thoracic kyphosis = T1 incidence − L1 incidence; C2–C7 lordosis = T1 incidence − C2 incidence; L1 slope = L1 incidence − pelvic tilt; T1 slope = T1 incidence − pelvic tilt; C2 slope = C2 incidence − pelvic tilt.

After training an algorithm that recognizes 11 points at the center of both femoral heads, endplates of the sacrum, L1, T1, C2, and the center of the sacrum for 500 people, the pelvic parameters of pelvic incidence (PI), pelvic tilt (PT), and sacral slope (SS), along with three incidence angles of L1, T1, and C2, were measured using a self-made program. The six test parameters of lumbar lordosis (LL), thoracic kyphosis (TK), C2–C7 lordosis (C2–7 L), L1S, T1S, and C2S were calculated using the following equations using the concept of IAIPs. Table 1 and Figure 4 present the definitions and calculation equations for the sagittal parameters.

All parameters were defined as negative for lordosis and positive for kyphosis to ensure the uniformity of measurements. The incidence angle was defined as positive when the perpendicular line of the corresponding endplate was located on the right side compared with the PT line, similar to the pelvic incidence, and negative for the left side. The slope angle was defined as positive for the right upside open angle, such as the sacral slope, and negative for the left upside open angle.

## 5. Validation Process

In addition to the training set of 500 individuals, whole-spine standing radiographs of 63 individuals who had not been included in the training set were used for the validation process. Each spinal sagittal parameter was manually measured twice at 2-week intervals by two spine surgeons. The mean values were employed as the established reference standards. To assess the accuracy of the parameters measured by machine learning, the success rate was determined using a Bland–Altman plot. Success rates were defined as the proportion of cases where the mean absolute error, as measured by the CNN, fell below a predefined error threshold.

To evaluate the accuracy of the proposed equation, the concordance between the direct measurement values of the six test parameters and the values derived from the calculated equation was analyzed. Concordance was analyzed by comparing the mean and standard deviation (SD) error, Pearson correlation coefficient, and coefficient of determination. Interobserver reliability and intraobserver reproducibility were analyzed using intraclass correlation coefficients (ICCs).

## 6. Results

**Figure 5:**
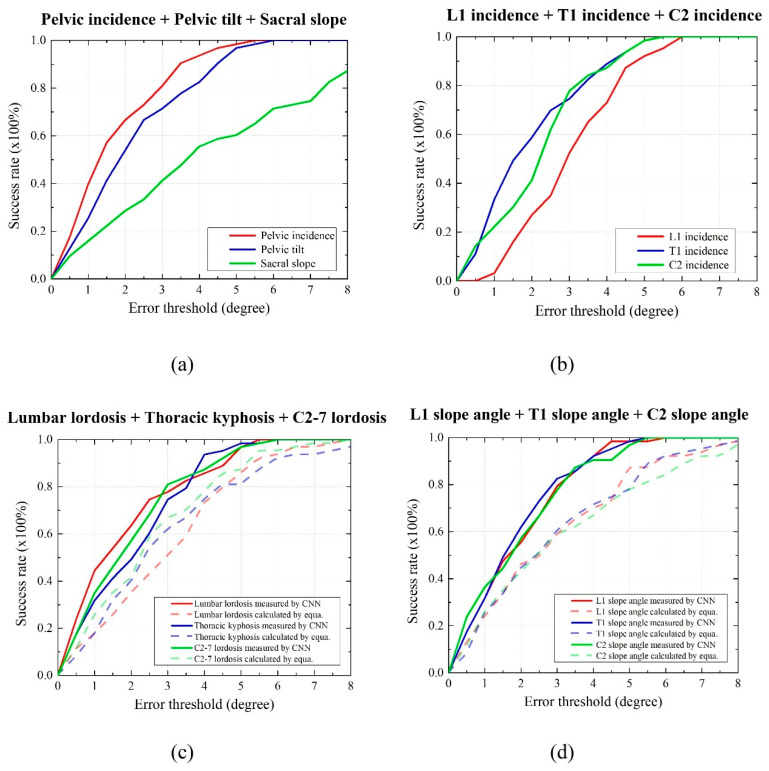
Success rates for 12 parameters by error threshold; (**a**) pelvic incidence, pelvic tilt, and sacral slope angles measured by CNN; (**b**) L1 incidence, T1 incidence, and C2 incidence angles measured by CNN; (**c**) lumbar lordosis, thoracic kyphosis, and C2–C7 lordosis angle measured by CNN and calculated from the equations; and (**d**) L1 slope, T1 slope, and C2 slope angles measured by CNN and calculated from the equations.

The descriptive data of the CNN and standard reference values for the 63 validation sets are listed in Table 2. The success rates of the parameters were high for PI and C2I, and low for SS and L1I (Figure 5).

The proposed method demonstrated notable efficiency in measuring various parameters, including PI, PT, T1I, C2I, LL, C2-7 L, L1S, T1S, and C2S, achieving a detection rate of 80%, at an error threshold of 3.5° for these angles. Furthermore, visualized results exhibited distinct characteristics when compared to the remaining outcomes, particularly in cases where they achieved a 0.8 detection rate at an error threshold of 7.5° for SS and 4.5° for L1I.

**Figure 6:**
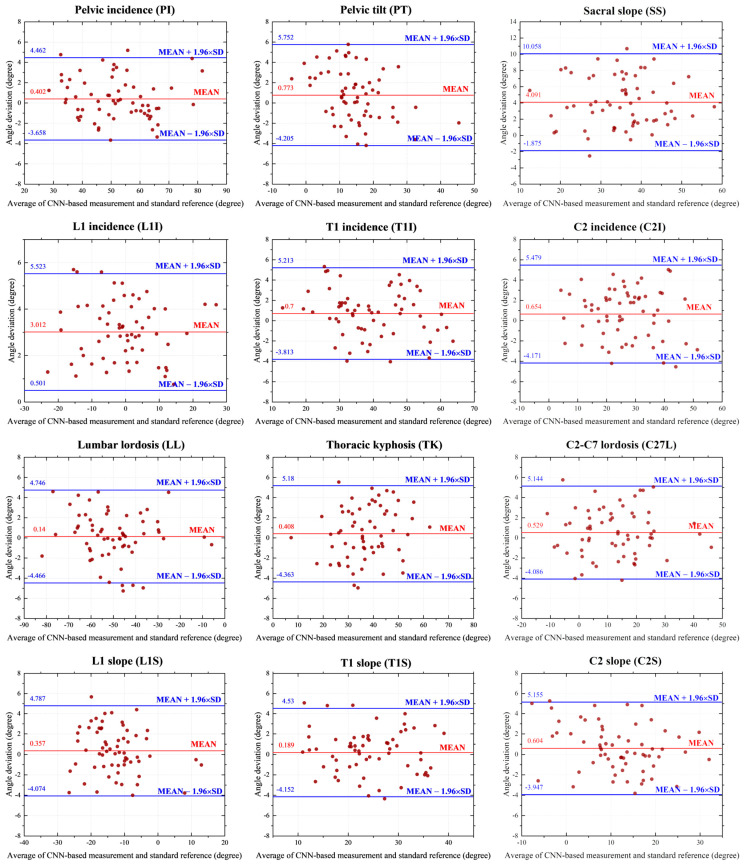
Bland–Altman plots comparing values of radiographic parameters.

The test results of our method were graphically represented through Bland–Altman (B-A) plots, as depicted in Figure 6. These plots include horizontal lines indicating the mean difference (represented by the red solid line) and the mean difference ± 1.96 standard deviations (indicated by the blue dashed lines). Remarkably, the entire measurement process required less than 1 s to complete.

Table 3 and Table 4 present the mean and standard error of the absolute differences between the values computed using the proposed equation and the corresponding standard reference values obtained via the CNN method for the six test parameters. The mean absolute differences between the calculated values and those directly measured by the CNN were 0.045 in L1S, T1S, and C2S; and 0.006 in LL, TK, and C2-7 L, with a coefficient of determination of 1.0 (*p* < 0.01). The mean absolute difference value between the value calculated by CNN and the standard reference was 1.8, with a coefficient of determination of 0.96 (*p* < 0.01). All test parameters showed statistically significant ICC value over 0.9 with statistical significance (Table 5, *p* < 0.01).

## 7. Discussion

This study conducted a machine learning-based geometric analysis of global spinal alignment. The algorithms were able to accurately detect 11 key anatomical landmarks, such as the center of the femoral head and sacrum upper endplate, each endpoint of the superior sacrum, L1, T1 superior endplate, and C2 inferior endplate. CNNs are the most optimized method for image learning, and are being actively studied in the field of imaging medicine. In an early study on the analysis of spinal sagittal parameters using machine learning, Zhang et al. [28] automatically measured the Cobb angle in patients with scoliosis. When 235 of 340 simple radiographic examinations were subjected to machine learning and 105 were tested, the difference between the artificial neural network and manual measurement by the spinal surgeon was more than 5°. Similarly, Galbusera et al. [29] reported the results of machine learning for measuring thoracic kyphosis of T4–T12, lumbar lordosis, Cobb angle, pelvic incidence, sacral slope, and pelvic tilt based on a fully convolutional neural network. After training on data from 443 individuals and subsequent evaluation by 50 individuals, it was observed that all predicted parameters exhibited a robust correlation with values assessed by experienced spine surgeons. The standard error of the estimated parameters varied from 2.7° for the pelvic tilt angle to 11.5° for the lumbar spine.

Recently, Yeh et al. [30] also published a convolutional neural network model that detected 45 anatomical landmarks and measured major sagittal alignment parameters using sagittal radiographs. The accuracy was improved using 2200 evaluation datasets and was related to the number of detection points of the anatomical indicators. That is, the accuracy of the parameters of the cervical and lumbar vertebrae composed of two anatomical indicators was high, whereas the accuracy of the thoracic and pelvic indicators composed of four indicators difficult to distinguish from the structures around the thoracic vertebrae in the thoracic vertebrae was relatively low. The performance of the convolution model was highest in the cervical vertebrae, and the error ranged from 1.75 to 2.64 mm, followed by the lumbar vertebrae, with an error ranging from 1.76 to 2.63 mm. However, the thoracic region showed a larger error, and the error range was 2.21~3.07 mm; the error of the pelvic incidence was the highest, and the central error for the measurement of the center of the femoral head was 3.39 mm. In their study, Yeh et al. localized 45 anatomical landmarks; however, many of them were considered to have contributed to increasing error. In addition, the localization error in finding the center of the femur head was the greatest among all the anatomic landmarks, and the error distribution was the widest; therefore, the accuracy of the pelvic parameters was low in their study.

The decentralized CNN employed in this study offers a decentralized approach across multiple detection orders, enabling the attainment of high accuracy, even with a limited number of training images—an aspect of paramount significance in the realm of medical metrology. Based on the conventional CNN model structure for locating a landmark’s horizontal and vertical positions, the developed method utilizes multiple trained CNN models intentionally arranged in order. In increasing order, the landmark positions are predicted from rough to precise accuracy, which consequently provides sufficient final detected positions of the considered vertebrae, as well as good measured angles. This advantage comes from the procedure of narrowing the ROI according to each order, which not only reduces the number of unrelated features that can affect the results, but also increases the diversity of the training dataset.

In this study, six geometrical equations were proposed to efficiently measure the angular parameters from three pelvic parameters and the IAIPs. The accuracies of the six test parameters were 99% between the measured values and those calculated by the spine surgeons. For the manual measurement of a specific angular parameter, the observer must set two vectors and measure the Cobb angle between them. Therefore, 24 vectors must be obtained to measure 12 parameters, and measurement errors can occur during this process. However, 12 angular parameters could be measured with high accuracy using only 11 key anatomical landmarks, machine learning, and the equations proposed in this study. In addition, lordosis of the cervical and lumbar spines should be defined as negative; however, in clinical settings, absolute values are used with confusion. These mistakes can occur frequently in parameters with ranges including zero, such as the L1 incidence, L1 slope, C2–7 lordosis, and C2 slope. If the orientational definition of angular parameters is not accurately defined, large-scale data may be too heterogeneous to interpret the sagittal alignment. Disorganized data increases the entropy of the data, and this can be a confounding factor in revealing the characteristics of spinal sagittal alignment. Using the orientational definition of the slope and incidence angles, we collected homogeneous data with high accuracy. The authors believe that the definitions proposed in this study will be effective for large-scale data collection on spinal sagittal parameters.

The ICC values, as observed in this study, demonstrated a high level of accuracy in the detection of anatomical points by the CNN when compared to direct measurements by human experts. However, it is notable that as the detection rate of anatomical landmarks located in regions overlapping with the rib cage and shoulder girdle diminished, the ICC values for parameters such as T1 slope and T1 incidence exhibited reduced accuracy. Consequently, we acknowledge the need for further algorithmic enhancements to improve generalizability. This could entail adjustments in image contrast or substituting the T1 slope with the measurement of C7 slope.

Additionally, it should be noted that the algorithm for assessing distance parameters could not be fully evaluated in this study, and ongoing efforts are dedicated to its refinement. Lastly, we recognize the potential limitation posed by the use of spine images collected solely from a single institution, which may restrict external validation. Therefore, there is a compelling need for future algorithmic enhancements based on the incorporation of multicenter image data to ensure broader applicability and robustness.

## 8. Conclusions

The CNN-based deep learning algorithm and the concept of IAIPs were able to accurately measure the spinal sagittal parameters. Based on the three pelvic parameters and three incidence angles used, six additional parameters were accurately estimated. The advantages of machine learning and the concept of IAIPs shown herein suggest their utility for large-scale data accumulation in sagittal spinal alignment.

## Figures and Tables

**Table 1:** Definitions of radiographic parameters.

	Parameters	Definition
**Pelvic parameters**	**Pelvic incidence**	The angle between a line drawn from the center of the femoral heads to the midpoint of the sacral superior endplate and a line perpendicular to the sacral superior endplate.
	**Pelvic tilt**	The angle between a line drawn from the center of the femoral heads to the midpoint of the sacral superior endplate and the vertical line.
	**Sacral slope**	The angle between the sacral superior endplate and the horizontal line.
**Incidence angle**	**C2 incidence**	The angle between a line from the center of the femoral heads to the midpoint of the sacral superior endplate and a line perpendicular C2 inferior endplate.
	**T1 incidence**	The angle is measured as the deviation between a line drawn from the center of the femoral heads to the midpoint of the sacral superior endplate and a line that is perpendicular to the superior endplate of the T1 vertebra.
	**L1 incidence**	The angle is defined as the angle formed between a line extending from the center of the femoral heads to the midpoint of the sacral superior endplate and a line that is perpendicular to the superior endplate of the L1 vertebra.
**Test parameters**	**Cervical lordosis**	The angle between the inferior endplate of C2 and the superior endplate of T1.
	**C2 slope**	The angle between the horizontal and inferior endplate of C2.
	**Thoracic kyphosis**	The angle between the superior endplate of T1 and the superior endplate of L1.
	**T1 slope**	The angle between the horizontal and superior endplate of T1.
	**Lumbar lordosis**	The angle between the superior endplate of L1 and the superior endplate of S1.
	**L1 slope**	The angle between the horizontal and superior endplate of L1.

**Table 2:** Descriptive data for the radiographic measurements of sagittal parameters.

Parameters	CNN (*n* = 63)			Standard Reference (*n* = 63)		
	Mean ± SD	Min.	Max.	Mean ± SD	Min.	Max.
**Pelvic incidence (°)**	52.16 ± 12.24	29.14	83.31	51.76 ± 12.47	27.94	80.18
**Pelvic tilt (°)**	14.99 ± 8.03	−3.00	44.55	14.21 ± 8.94	−5.36	46.55
**Sacral slope (°)**	37.22 ± 9.40	15.20	59.95	33.13 ± 9.42	9.69	56.46
**L1 incidence (°)**	2.46 ± 13.05	−22.42	55.76	−0.56 ± 13.3	−23.69	54.78
**T1 incidence (°)**	39.77 ± 11.33	13.73	62.78	39.07 ± 11.74	12.50	64.85
**C2 incidence (°)**	26.87 ± 11.03	4.53	50.15	26.21 ± 11.26	2.76	53.06
**Lumbar lordosis (°)**	−49.71 ± 14.34	−82.82	−6.05	−49.85 ± 14.63	−80.98	−5.34
**Thoracic kyphosis (°)**	37.30 ± 10.31	7.02	61.62	36.89 ± 10.53	7.62	63.71
**C2–C7 lordosis (°)**	12.89 ± 12.32	−9.82	45.84	12.37 ± 12.37	−12.21	46.81
**L1 slope (°)**	−12.48 ± 7.80	−28.37	12.60	−12.84 ± 8.22	−25.34	13.66
**T1 slope (°)**	24.82 ± 7.71	11.06	44.80	24.64 ± 8.04	8.76	48.34
**C2 slope (°)**	11.93 ± 8.37	−7.58	31.87	11.32 ± 9.05	−10.17	32.40

**Table 3:** Comparison of radiographic parameters between computed and measured values using convolutional neural networks.

Parameters	Calculated Value by CNN (*n* = 63)	Measured Value by CNN (*n* = 63)	Absolute Difference Value		Correlation Coefficient	Coefficient of Determination	*p*-Value
	Mean ± SD	Mean ± SD	MAE (°)	STD of AE (°)			
**Lumbar lordosis (°)**	−49.71 ± 14.34	−49.71 ± 14.34	0.004	0.005	1.000	1.000	0.000
**Thoracic kyphosis (°)**	37.30 ± 10.31	37.31 ± 10.31	0.008	0.363	1.000	1.000	0.000
**C2–C7 lordosis (°)**	12.90 ± 12.32	12.90 ± 12.32	0.004	0.012	1.000	1.000	0.000
**L1 slope (°)**	−12.48 ± 7.80	−12.53 ± 7.80	0.046	0.005	1.000	1.000	0.000
**T1 slope (°)**	24.83 ± 7.71	24.78 ± 7.71	0.044	0.006	1.000	1.000	0.000
**C2 slope (°)**	11.93 ± 8.37	11.88 ± 8.37	0.045	0.006	1.000	1.000	0.000

**Table 4:** Comparison of radiographic parameters between calculated values by convolution neural networks and standard reference.

Parameters	Calculated Value by CNN (*n* = 63)	Measured Value of Standard Reference (*n* = 63)	Absolute Difference Value		Correlation Coefficient	Coefficient of Determination	*p*-Value
	Mean ± SD	Mean ± SD	MAE (°)	STD of AE (°)			
**Lumbar lordosis (°)**	−49.71 ± 14.34	−49.85 ± 14.63	1.764	1.543	0.987	0.974	0.000
**Thoracic kyphosis (°)**	37.31 ± 10.31	36.89 ± 10.53	2.019	1.399	0.973	0.947	0.000
**C2–C7 lordosis (°)**	12.90 ± 12.32	12.37 ± 12.37	1.938	1.437	0.982	0.964	0.000
**L1 slope (°)**	−12.53 ± 7.80	−12.84 ± 8.22	1.843	1.326	0.961	0.924	0.000
**T1 slope (°)**	24.78 ± 7.70	24.64 ± 8.04	1.773	1.315	0.961	0.924	0.000
**C2 slope (°)**	11.88 ± 8.37	11.32 ± 9.05	1.882	1.454	0.967	0.936	0.000

**Table 5:** Intraclass correlation coefficient values of six test parameters.

	ICC (2,1)	95% CI	*p*-Value
**Pelvic incidence**	0.993	0.988–0.996	0.000
**Pelvic tilt**	0.975	0.958–0.986	0.000
**Sacral slope**	0.928	0.217–0.979	0.000
**L1 incidence**	0.985	0.203–0.997	0.000
**T1 incidence**	0.989	0.981–0.994	0.000
**C2 incidence**	0.987	0.978–0.992	0.000

## Data Availability

The data used in this study is not available for public sharing due to privacy and confidentiality considerations.
